# Mechanical insufflation-exsufflation to promote extubation success in critically ill adults on intensive care: protocol for a randomised controlled feasibility trial

**DOI:** 10.1186/s40814-023-01362-7

**Published:** 2023-07-24

**Authors:** Ema Swingwood, Sarah Voss, Lyvonne N. Tume, Jeremy Bewley, Nicholas Turner, George Ntoumenopoulos, Louise Rose, Fiona Cramp

**Affiliations:** 1 Faculty of Health and Applied Sciences, University of the West of England, Bristol, UK; 2 Adult Therapy Services, University Hospitals Bristol and Weston NHS Foundation Trust, Bristol, UK; 3 Faculty of Health, Social Care & Medicine, Edge Hill University, Ormskirk, UK; 4 Department of Intensive Care, University Hospitals Bristol and Weston NHS Foundation Trust, Bristol, UK; 5 Bristol Trials Centre, Bristol Medical School, University of Bristol, Bristol, UK; 6 Department of Physiotherapy, St Vincent’s Hospital, Sydney, Australia; 7 Florence Nightingale Faculty of Nursing, Midwifery and Palliative Care, King’s College London, London, UK; 8 Department of Critical Care and Lane Fox Respiratory Unit, Guy’s and Thomas’ Foundation NHS Hospital Trust, London, UK

**Keywords:** Cough assist, Extubation failure, Ventilator weaning, Physiotherapy, ICU, Airway clearance, Electrical impedance tomography

## Abstract

**Background:**

Extubation failure, defined as reintubation within 48 h, is associated with increased intensive care unit (ICU) length of stay and higher mortality risk. One cause of extubation failure is secretion retention, resulting from an inability to cough effectively. Mechanical insufflation-exsufflation (MI-E) simulates a cough aiding secretion clearance. However, MI-E is not routinely used in the ICU for invasively ventilated patients. This study aims to determine feasibility and acceptability of a randomised controlled trial (RCT) examining MI-E use to promote extubation success in intubated, ventilated adults.

**Methods:**

It is a single-centre, feasibility RCT with semi-structured interviews, economic scoping, and exploratory physiology study.

The feasibility RCT (*n* = 50) will compare standard care to a MI-E protocol including a minimum of two MI-E sessions via the endotracheal tube prior to extubation. Post-extubation, MI-E will be delivered via facemask or mouthpiece up to two times/day for 48 h. MI-E settings will be individualised. All patients will receive standard care (no MI-E) in relation to mechanical ventilation, weaning, rehabilitation, physiotherapy techniques such as positioning, manual airway clearance techniques, manual/ventilator hyperinflation, endotracheal suctioning, and nebulisation. Clinical data collection will occur before, on completion, and 5-min post-physiotherapy sessions (intervention/control arms). Resource use will be calculated for each 24-h period. Analyses will be descriptive and address feasibility outcomes including participant recruitment and attrition, proportion of MI-E treatment sessions completed, dataset completeness, and frequency of adverse events and acceptability.

Semi-structured online interviews informed by the Theoretical Framework of Acceptability (TFA) with patients, clinicians, and family members will explore the acceptability of the MI-E intervention and study processes.

Interview data will be analysed using reflexive thematic analysis based on TFA domains through first-level coding.

The embedded physiology study will use electrical impedance tomography and lung ultrasound to explore lung recruitment and de-recruitment during MI-E in a subset of 5–10 patients.

**Discussion:**

This study will examine feasibility and acceptability of a RCT protocol of MI-E to promote extubation success. Study findings will inform design modification and conduct of a future adequately powered trial. Furthermore, the study will contribute and advance the understanding of MI-E use in critically ill intubated adults.

**Trial registration:**

ISRCTN 24603037; IRAS 303674

**Supplementary Information:**

The online version contains supplementary material available at 10.1186/s40814-023-01362-7.

## Background

Extubation failure is defined as reintubation within 48 h and is associated with increased intensive care unit (ICU) length of stay (LOS) [1] and higher mortality risk [2]. One cause of extubation failure is secretion retention, resulting from an inability to cough effectively [3]. Having an endotracheal tube in place impairs the ability to cough due to abduction of the vocal cords and glottis. As a result, airway clearance strategies are used to aid secretion clearance. Suctioning is used commonly to remove secretions from the endotracheal tube, tracheostomy, or upper airway. This technique however has limited effectiveness in clearing secretions from the lower airways and may cause airway trauma [4, 5].

Mechanical insufflation-exsufflation (MI-E) augments inspiratory and expiratory flow to improve secretion mobilisation, through rapidly alternating positive and negative pressure, approximating a normal cough [6]. A previous randomised controlled trial (RCT) based in Portugal examined MI-E in 75 critically ill adults intubated for > 48 h [7]. Using MI-E, they found reductions in re-intubation rates (48% v 17%), mechanical ventilation duration (mean (SD) 17.8 [6] v 11.7 (3.5) days), and ICU LOS post-extubation (9.8 (6.7) v 3.1 (2.5) days (all *p* < 0.05)). More recent trials have demonstrated the superiority of MI-E compared to other airway clearance techniques on physiologic outcomes including sputum weight, static lung compliance, airway resistance, and work of breathing [8, 9]. Recent studies regarding the safety of MI-E in intubated patients indicate that adverse effects such as barotrauma, desaturation, atelectasis, and haemoptysis are rare and transient [10, 11]. However, to date, there is limited adoption of MI-E in ICU [12–14] and limited empirical evidence on its effectiveness [15]. MI-E may be safe and effective in intubated critically ill adults, but more data are required.

During invasive ventilation, positive pressure breaths are delivered followed by a passive expiration. In contrast, MI-E delivers both positive (insufflation) and negative (exsufflation) pressure breaths. Barotrauma and volutrauma associated with large tidal volumes are well documented, with low volume lung-protective ventilation now standard of care, particularly for patients with acute lung injury [16]. However, de-recruitment of lung units due to small tidal volumes can have an equally adverse impact on oxygenation and effective ventilation, attenuating lung injury [17]. To date, no studies have examined the extent of de-recruitment or other adverse events as a result of a negative pressure exsufflation breath applied during MI-E.

We recently conducted a scoping review [18] including 28 studies to map use of MI-E in invasively ventilated critically ill adults. We found MI-E was predominantly used in ICU patients with prolonged weaning from mechanical ventilation and difficulty with sputum clearance. Study populations did not always reflect the heterogeneous nature of invasively ventilated critically ill adults, with some studies enrolling cohorts limited to neuromuscular disease and spinal cord injury. We identified substantial variation in MI-E device settings, timing, and frequency of use across studies.

The recent scoping review [18] also identified a lack of specific qualitative data pertaining to patient and clinician experience of using MI-E. Information was gained through three survey studies which reported qualitative data from open-ended questions around barriers to MI-E in ICU. A common barrier to MI-E use was a perceived lack of skills and knowledge. There were no studies that included patients’ opinions or experiences of MI-E use.

This variation in how MI-E is used combined with uncertainty in terms of the evidence of effect on patient outcomes such as promoting weaning success, reducing extubation failure and safety, limits the ability to make practice recommendations and warrants further investigation. Therefore, the aim of this study is to determine the feasibility of a RCT of MI-E to promote extubation success for intubated, mechanically ventilated critically ill adults.

Our objectives are to determine trial feasibility based on the following feasibility end points:

1. Ability to recruit and retain the proposed 50 participants
2. Ability to collect outcome data (including follow up data) and to examine dataset completeness
3. Acceptability of the MI-E intervention from the perspectives of patients, family, and members of the interprofessional team including doctors, nurses, and physiotherapists.

## Methods

**Fig. 1:**
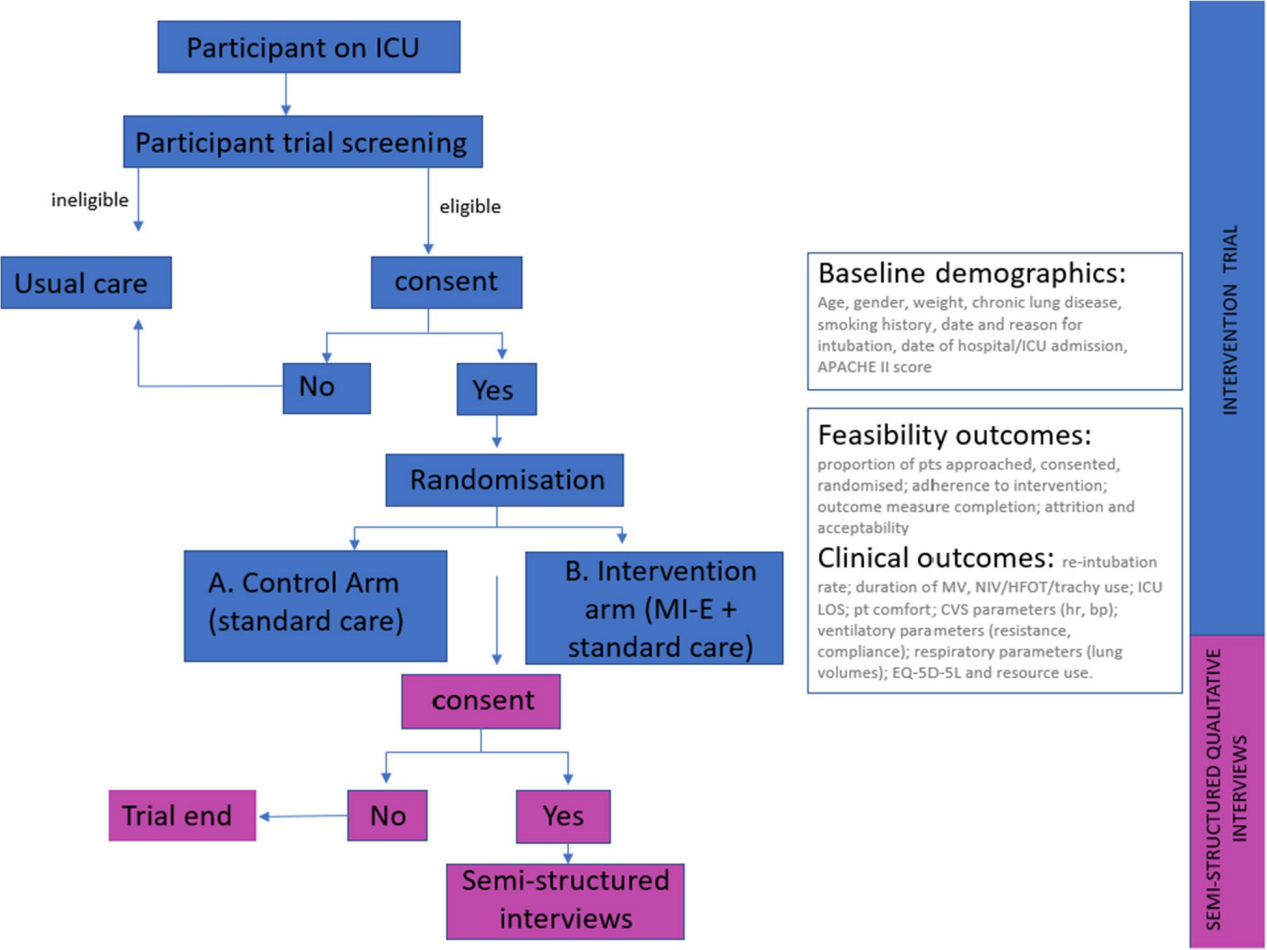
Study flow chart

The protocol conforms to the SPIRIT (Standard Protocol Items: Recommendations for Interventional Trials) guideline [19] and describes a single-centre, individual parallel group, randomised, feasibility RCT with semi-structured interviews, economic scoping, and the incorporation of an exploratory physiology study. A study flow chart is illustrated in Fig. 1; schedule for enrolment, intervention, and follow-up is shown in Table 1, with associated SPIRIT checklist presented in supplementary information 1.

**Table 1:** Schedule for enrolment, intervention, and follow-up

	**Data**	**Timepoint**						
		**Enrollment**	**Baseline**	**Pre-intervention**	**During intervention**	**5 min post intervention**	**Duration of study period**	**6-month follow-up**
**Enrolment**	Eligibility screening	X						
	Consent	X						
	Allocation	X						
**Intervention**	Standard care				X			
	MI-E intervention				X			
**Assessments: baseline demographic outcome**	Demographics (age, gender, predicted body weight, history of lung disease, smoking history)		X					
	Reason for intubation		X					
	Date of hospital and ICU admission		X					
	Date of intubation		X					
	Ventilator settings		X					
	Airway type and size		X					
	APACHE II score		X					
**Assessments: clinical outcomes**	Use of HFOT, NIV, tracheostomy						X	
	Use of physiotherapy interventions				X			
	LUS score			X		X		
	Patient pain/discomfort (CPOT; NRS)			X		X		
	CVS parameters (HR, SBP, DBP)			X		X		
	Ventilator parameters (vent settings, resistance, compliance)			X		X		
	Respiratory parameters (RR, SpO_2_)			X		X		
**Assessments: health economics**	• Resource use (staffing requirements, suction frequency, consumable use, antibiotic use, physiotherapy on-call use) • QoL (EQ-5D-5L)				X			X
**Assessments: safety**	Adverse events				X	X	X	

Abbreviations: *APACHE II* Acute Physiology and Chronic Health Evaluation, *CPOT* Critical Care Pain Observation Tool, *DBP* Diastolic blood pressure, *HFOT* High-flow oxygen therapy, *hr* Heart rate, *ICU* Intensive care unit, *LUS* Lung ultrasound, *NIV* Noninvasive ventilation, *NRS* Numeric rating scale, *SBP* Systolic blood pressure, *RR* Respiratory rate, *SpO2* Peripheral oxygen saturations, *QoL* Quality of life

### Feasibility RCT

The study will be conducted in a 21-bed general adult ICU, within a large UK National Health System (NHS) teaching hospital. The unit has approximately 1250 admissions annually and typically admits adults with any condition except cardiac or neurosurgery

#### Participant identification, recruitment, and allocation

##### Eligibility

A research team member will screen all ICU patients on a daily basis against the study eligibility criteria. Our inclusion criteria comprise the following:

- Adult (≥ 16 years)
- Expected to require invasive mechanical ventilation for > 48 h
- Clinician identified pre-extubation problems with secretion management defined as poor/weak cough effort and/or secretion load difficult to clear with usual airway clearance management, i.e. suctioning, manual techniques, and positioning (as assessed by the treating physiotherapy clinical team)
- Identified as ‘ready to wean or weaning’ by the treating clinical team and on a spontaneous mode of ventilation, for example continuous positive airway pressure (CPAP) or pressure support ventilation (PSV)

Our exclusion criteria comprise the following:

- Positive end-expiratory pressure (PEEP) > 10 cmH_2_O
- Fraction of inspired oxygen (FiO_2_) > 0.7
- Hemodynamic/cardiovascular instability as defined as noradrenaline infusion of > 0.25 mg/kg or arrhythmia requiring intervention
- Recent untreated pneumothorax (current admission with no chest drain in situ)
- Unable to use MI-E pre-/post extubation (contraindications to facemask use including facial/cranial trauma, recent facial surgery; active upper gastrointestinal bleeding/uncontrolled vomiting; recent upper abdominal/thoracic surgery with at risk anastomosis; acute air trapping, i.e. status asthmaticus)
- Pre-existing neuromuscular condition affecting respiratory muscles
- Pre-existing use of MI-E in the community
- Pre-existing permanent tracheostomy
- Treatment withdrawal expected within 24 h or not expected to survive
- Re-admission to ICU following index admission within same hospital episode
- Previous participation in the study

### Randomisation and allocation concealment

Using the online randomisation system ‘Sealed Envelope™’ (that conceals allocation), an ICU research team member will randomise a patient once informed consent/informed advice has been obtained and demographic data collected. Participants will be randomised using a 1:1 allocation to either (A)-control arm (standard care) or (B)-intervention arm (MI-E plus standard care). Blinding of participants, clinicians, and outcome assessors will not be possible due to the nature of the intervention.

#### Study arms

##### A. Control arm (standard care)

Patients will receive standard care in relation to mechanical ventilation, ventilator weaning, rehabilitation, standard physiotherapy techniques such as positioning, manual techniques (percussion, expiratory vibrations, expiratory shakes), manual/ventilator hyperinflation, endotracheal suctioning, and nebulisation. The use of MI-E will not be permitted in the standard care control arm. Respiratory physiotherapy treatments will be individualised to patient need at the discretion of the treating physiotherapist and not protocolised. Decisions to extubate and re-intubate will be at the discretion of the attending physician with reason(s) documented.

##### B. Intervention arm (MI-E plus standard care)

For the intervention arm, we will use the MI-E device, Clearway 2 (Breas Medical Ltd., Stratford-Upon-Avon, Warwickshire, UK). This device is reusable between patients with single-use circuit, filter, and interface (mouthpiece, facemask, and flexible catheter mount).

Whilst intubated, treatment will include a minimum of two MI-E sessions via the endotracheal tube (with cuff inflated) following randomisation and prior to extubation. MI-E settings (mode, pressure, timings, flow) will be individualised to each patient based on patient tolerance, chest expansion, and secretion clearance (as assessed by treating physiotherapist, see supplementary file 2). There will be no minimum/maximum time between MI-E sessions. Following extubation (and up to 48 h), patients will receive MI-E delivered via facemask or mouthpiece up to 2 times each day.

#### Outcomes

Feasibility outcomes are listed in Table 2. Clinical endpoints will be collected to understand the feasibility of their collection informing conduct of a future adequately powered trial and not to conduct hypothesis testing related to causation. Feasibility will be assessed using pre-defined progression criteria (Table 3).

**Table 2:** Feasibility outcomes

Feasibility outcome	Measurement detail
Proportion of eligible patients approached, consented, and randomised	Screening log and randomisation records
Proportion of MI-E treatment sessions completed	Case report form
Proportion of recruited patients with all clinical outcomes recorded	Case report form
Frequency of adverse events	Case report form
Attrition (participant withdrawal and loss to follow-up)	Case report form and withdrawal records
Acceptability of intervention and trial processes to participants and clinicians	Qualitative interviews Acceptability of intervention measure (AIM)/intervention appropriateness measure (IAM)/feasibility of intervention measure (FIM)
Acceptability of outcome measures to participants and clinicians	Qualitative interviews

**Table 3:** Progression criteria (based on feasibility parameters)

	**Summary**	**Action required**
Go (green)	**Recruitment**: > 70% expected recruitment target **Follow-up**: > 75% data completeness **Adherence**: > 75% adherence to intervention	Feasible to continue to main trial
Amend (amber)	**Recruitment:** 50–70% of expected recruitment target **Follow-up**: 65–75% data completeness **Adherence**: 65–75% adherence to intervention	Identify remediable factors; discuss with trial management group
Stop (red)	**Recruitment**: < 50% of expected recruitment target **Follow-up**: < 65% data completeness **Adherence**: < 65% adherence to intervention	Do not progress to main trial, unless there is a strong case that unanticipated remediable factors have been identified

#### Data collection

Prior to randomisation, the research team will collect baseline demographic and clinical characteristic data from the electronic medical record. Data include general demographics, reason for intubation, date of hospital and ICU admission, date of intubation, admission Acute Physiology and Chronic Health Evaluation (APACHE II), baseline ventilator settings, and airway type and size (Table 1).

Clinical outcomes (Table 1) will be measured before, on completion, and 5 min after physiotherapy sessions for both study arms. We have selected exploratory clinical outcomes using the core outcome measure set for critical care ventilation trials [20]. In addition, we will record the number and type of physiotherapy treatments provided, patient pain/discomfort, cardiovascular parameters, ventilatory parameters, and respiratory parameters (see Table 1 for further details).

To assess the feasibility of collecting data for a cost-utility analysis in a future trial, we will collect the following:

- EQ-5D-5L at 6-month post-ICU discharge
- Resource use associated with the MI-E intervention and standard care

We will identify the following resource use during the index admission: MI-E device-associated resource use including staffing requirements (time spent delivering an MI-E treatment, grade/seniority of staff administering treatment) and consumables used. Patient-related resource use will include endotracheal suction frequency by nursing staff (over a 24-h period), use of noninvasive ventilation (NIV), high-flow oxygen therapy (HFOT) and tracheostomy, antibiotic use, physiotherapy on-call use (planned and unplanned), ICU LOS, ICU re-admission and hospital LOS. For the purposes of the feasibility trial, these will be reported as frequencies and time duration (hours).

### Clinician training

Training for physiotherapists detailing the study protocol and how to deliver the intervention will occur at the start of the study. Standardised education materials developed by the research team will be distributed to all clinicians with the opportunity to practice intervention set up and delivery.

### Outcome description

- *Re-intubation rate*: Re-intubation rate will be calculated for the 48 h following extubation. This is the planned primary outcome for the future planned trial.
- *Pain scores*: We will measure pain using the ‘numeric rating scale’ (NRS) [21] and the Critical Care Pain Observation Tool (CPOT) [22]. All patients will have CPOT measured. The CPOT is a valid measure to determine pain presence with four domains: facial expressions, body movements, compliance with the ventilator or vocalisation, and muscle tension. Each domain is scored 0–2 with a maximum score of eight. A CPOT score > 2 indicates pain presence. The NRS is a self-reported measure where patients rate pain presence and severity on a scale from 0 (no pain) to 10 (worst pain possible). During PPI work, patients highlighted the importance of including a patient-reported outcome. The NRS will be measured in addition to the CPOT. If a patient is unable to rate pain, we will use the CPOT only. We will document pain presence before and after a physiotherapy session.
- *Cardiovascular, ventilator, and respiratory parameters*: These measures include heart rate, systolic and diastolic blood pressure, ventilator settings, airway resistance and lung compliance, peripheral oxygen saturations, and respiratory rate measured pre- and post physiotherapy in both the intervention and control arms.
- *Acceptability*: We will use three validated questionnaires to measure acceptability: acceptability of intervention measure (AIM), intervention appropriateness measure (IAM) and feasibility of intervention measure (FIM) [23]. These will be measured immediately post-MI-E intervention.

#### Statistics and data analysis

##### Sample size calculation

As this is a feasibility trial, a formal sample size calculation based on statistical power to detect a specified treatment effect size is not appropriate. We have selected a sample size of 50 participants based on measurement of feasibility parameters with adequate precision. The participating ICU admits approximately 1250 patients annually with potentially four to five eligible patients each week (minimum of 200 per year). We anticipate recruiting 50 over a 12-month period would be achievable, with an estimated recruitment rate of 25% and a confidence interval width of 0.12.

##### Statistical analysis plan

The analysis and reporting of this study will be consistent with the CONSORT guidelines extension to feasibility studies [24]. This study is not designed or powered to carry out formal hypothesis testing. Participant flow through the study will be summarised and presented in a flow diagram. Descriptive statistics for patient characteristics will be reported overall and by treatment group: as means or medians with measures of dispersion for continuous outcomes (as appropriate given distribution) and frequencies and percentages for categorical outcomes. Only descriptive statistics will be used in the physiology sub-study due to the small sample size proposed. Patient-reported and clinical feasibility outcomes will be presented and assessed for completeness of data.

##### Safety reporting

The attending consultant physician is responsible for assessing all adverse reactions and adverse events (AEs) and categorising seriousness, expectedness, and relatedness. A list of events that can be expected during this trial, or within this patient population, can be found below.

- Accidental extubation during the intervention
- Cardiovascular changes (including but not exclusive to hypo/hypertension, brady/tachycardia, arrhythmias)
- Pneumothorax
- Sputum plugging during the intervention
- Pulmonary complications such as pneumonia
- Minor skin irritations due to electrical impedance tomography electrode patch application.

We will record occurrence of the following during a MI-E treatment and control arm interventions: HR, SBP, and DBP increase/decrease > 20% baseline and requiring intervention, arrhythmia (requiring intervention), pneumothorax, acute desaturation to < 85% or > 10% below baseline and requiring intervention, accidental extubation, and cardiopulmonary arrest.

It is the responsibility of the sponsor, chief investigator, and delegated individuals to ensure that the dignity, rights, safety, and well-being of research participants are given priority at all times, and appropriate action is taken to ensure their safety. The recording and reporting of safety events will be in accordance with good clinical practice (GCP) guidelines and study sponsor’s ‘research safety reporting’ standard operating procedure.

### Semi-structured qualitative interviews

Interviews with healthcare professionals and patients will explore the acceptability of the intervention and enrolment to the study. These interviews aim the following:

- Explore acceptability of the intervention for clinicians, patients, and consultees.
- Investigate potential barriers and facilitators to conducting a full trial.
- Determine outcome measures for a definitive trial.

#### Study design and recruitment

Interviews with patient participants in the intervention arm and their family members will take place within 6 weeks of discharge from ICU. We will exclude participants who have no recall of their ICU stay or the MI-E intervention. Interviews will be conducted by the chief investigator (E. S.).

Clinician interviews will be conducted with staff from the ICU clinical team including doctors, nurses, and physiotherapists who have had exposure to the MI-E intervention within the preceding 4 weeks. These interviews will be completed by a member of the study team (SV) to eliminate potential bias presented due to a working relationship with ES. These will occur during trial recruitment and within 4 weeks of exposure to a patient in the intervention arm of the trial.

We have based the interview topic guides on the Theoretical Framework of Acceptability (TFA) [25]. Interviews will be completed virtually via an online platform (Microsoft Teams).

#### Sampling and recruitment

Convenience sampling of 10–15 participants [26] will be used. Clinicians will be approached based on gaining maximal variation sample regarding profession and years of clinical experience. Patients and family members recruited into the study will be approached for consent once the patient has been discharged from ICU.

#### Interview data collection and analysis

On interview commencement, we will collect clinician demographic data (clinical profession, years working in profession and on ICU, highest educational level obtained) and patient demographics including age, reason for ICU admission, ICU LOS, or family demographics (relationship to patient) as relevant to the interview participant.

Interviews will be digitally recorded and transcribed verbatim by a university-approved transcription service. Transcripts will be checked for accuracy and anonymised. Data will be analysed using reflexive thematic analysis [26, 27] and using TFA domains through first-level coding by ES. Thematically similar responses will be grouped in a process of data reduction and compared across transcripts. Tables will be produced to highlight key thematic content, within each TFA domain with consideration of responses from both patients and clinicians and with the aim of highlighting similar and discordant themes. Domains will be identified as salient based on their frequency of inclusion and potential strength of impact. NVivo software will be used to support this process.

### Embedded exploratory physiology study

#### Background

During invasive ventilation, positive pressure breaths are delivered followed by passive expiration. In contrast, MI-E delivers both positive (insufflation) and negative (exsufflation) pressure breaths. Lung recruitment and de-recruitment are important considerations in intubated and ventilated patients [16]. Barotrauma and volutrauma associated with large tidal volumes are well documented, with low volume lung-protective ventilation now standard of care, particularly for patients with acute lung injury. De-recruitment of lung units due to small tidal volumes and loss of PEEP through ventilator disconnection can have an equally adverse impact on oxygenation and effective ventilation, attenuating lung injury [16].To date, no studies have examined the extent of recruitment and de-recruitment as a result of positive and negative pressure delivery during MI-E application.

#### Sub-study aim

To examine lung recruitment and de-recruitment during MI-E application.

#### Sub-study design

We will use electrical impedance tomography (EIT) (PulmoVista 500, Draeger Medical UK Ltd., Hertfordshire, UK) and lung ultrasound (Venue Go™, GE Healthcare, London, UK) in a subset of patients in the intervention arm. We aim to recruit between five and ten patients.

EIT is a noninvasive, radiation-free technique used at the bedside to provide pulmonary ventilation data in real time [28]. A series of 16 electrodes are placed around the chest wall, through which small electrical currents are passed to measure impedance, conductivity, and permittivity. These measurements result in a 2D image illustrating end inspiratory and end expiratory lung volumes and regional distribution of ventilation. The technique is used clinically and in ICU research studies to examine ventilation strategies, PEEP titration, and effects of positioning [28, 29].

#### Lung ultrasound score (LUS)

The lung ultrasound score is a semiquantitative scoring method used to illustrate pulmonary aeration [30]. We will use the previously described framework for practical application of the LUS in the ICU [31]. The framework describes six areas of interest per lung. Each hemithorax is divided into anterior, lateral, and posterior regions with each region having an upper and lower position. There is one representation point per area scanned and scored between 0 and 3 as part of this framework. Total scores range between 0 and 36. We will calculate LUS score pre- and post intervention. Scans will be completed by a clinician with Focused Ultrasound in Intensive Care (FUSIC) accreditation.

#### Data collection and reporting

We will record end-inspiratory and end-expiratory regional ventilation distribution via EIT before, during, and 5 min after the MI-E intervention. The lung ultrasound score will be calculated before and after the MI-E intervention (Table 3). Results will be presented as a case series.

#### Consent

On initial trial enrolment, patients may lack capacity to provide informed consent. As permitted in the UK, we will use a personal or nominated professional consultee. On regaining capacity, the patient will be informed of trial participation, and informed consent will be sought.

Interview participants will be requested to provide consent at the point of recruitment. Verbal informed consent will also be sought and recorded at the start of each interview.

#### Study withdrawal and processes

Participants are free to withdraw from any element of the study at any time without providing a reason. Unless specifically stated by the individual, data collected up to that point will be retained for analysis.

#### Data management

All participants will be assigned a unique study identification number, which will be used in all study-related documentation. A record of names, contact details, hospital numbers, and assigned trial numbers will be stored securely using a password-protected Research Electronic Data Capture (REDCap) database only accessible to members of the research team.

Clinical study data will be inputted directly into REDCap by the treating clinician and subsequently validated by a member of the research team. Study participants completing an online EQ-5D-5L survey will enter data directly through an external user REDCap interface. Data recorded on paper will be entered into the REDCap database (by E. S.).

Password-protected audio digital recording of interviews will be uploaded to a university computer secure drive. All transcriptions will be labelled with a unique study identification number, edited to ensure respondents are pseudonymised (only clinician profession and banding/grading documented), and stored securely adhering to university data protection policies.

Consent forms (and any other documentation) with personal identifiable data will be stored in a locked filing cabinet (or locked equivalent). Participant details will be anonymised in any publications that result from the trial. At the end of the study, pseudonymised data will be stored in a secure research data storage repository, alongside the other study data (as per sponsor policies).

#### Study management

A Trial Management Group (TMG) will be responsible for overseeing day-to-day study management. The TMG will meet weekly. We formed a 12-member patient advisory group (PAG) who have informed decisions related to study design and will have ongoing input into study conduct, data analysis, and interpretation and dissemination. Two PAG members will also participate in the Trial Steering Group (TSG) to ensure the patient voice is heard throughout the study. The TSG consists of 5 expert clinicians representing the ICU multi-professional team and has an independent chair. The group meet every 3 months during study conduct.

## Discussion

This study will investigate the feasibility of a RCT examining the use of MI-E to promote extubation success in critically ill adults receiving invasive mechanical ventilation. The importance and potential usefulness of completing a feasibility trial are further emphasised when considering the variability in MI-E use in intubated adults and variable outcome reporting as described in our recent scoping review [18]. The lack of qualitative data highlighted in the scoping review will be addressed in this trial through the completion of semi-structured interviews with clinicians, patients, and families. Additionally, the nested physiology study using EIT and LUS will provide a novel insight into the physiological impact of the MI-E device on lung recruitment and de-recruitment. Through the use of both quantitative and qualitative findings, we aim to optimise the design of a definitive trial particularly in relation to intervention and study protocol acceptability whilst also contributing and advancing the understanding of MI-E use in the acutely intubated population.

### Trial status

Recruitment commenced on 11th July 2022. The current protocol version (v2.0) is dated 21st March 2022. Recruitment is estimated to be complete by July 2023.

## Supplementary Information

**Additional file 1: **SPIRIT 2013 Checklist: Recommended items to address in a clinical trial protocol and related documents*.

**Additional file 2: Appendix 1.** Intervention arm protocol.

## Data Availability

This data will be made available in any form to those outside the trial, to include requirements of inspection purposes by the sponsor and/or other regulatory authorities. Individuals interested in study materials may contact the study CI (E. S.).

## Abbreviations

AE: Adverse events
AIM: Acceptability of Intervention Measure
APACHE II: Acute Physiology and Chronic Health Evaluation
ASB: Assisted Spontaneous Breathing
CONSORT: Consolidated Standards of Reporting Trials
CPAP: Continuous Positive Airway Pressure
CPOT: Critical Care Pain Observation Tool
DBP: Diastolic Blood Pressure
EIT: Electrical Impedance Tomography
FiO_2_: Fraction of Inspired Oxygen
FIM: Feasibility of Intervention Measure
FUSIC: Focused Ultrasound in Intensive Care
GCP: Good Clinical Practice
HR: Heart Rate
HFOT: High-flow Oxygen Therapy
IAM: Intervention Appropriateness Measure
ICU: Intensive Care Unit
LOS: Length of Stay
LUS: Lung Ultrasound Score
MI-E: Mechanical Insufflation-Exsufflation
NHS: National Health Service
NIV: Noninvasive ventilation
NRS: Numeric rating scale
PAG: Patient advisory group
PEEP: Positive end-expiratory pressure
REDCap: Research Electronic Data Capture
RCT: Randomised controlled trial
SBP: Systolic blood pressure
SPIRIT: Standard Protocol Items: Recommendations for Interventional Trials
TFA: Theoretical Framework of Acceptability
TMG: Trial Management Group
TSG: Trial Steering Group
UK: United Kingdom
