# Supplementary material for: Mechanical insufflation-exsufflation to promote extubation success in critically ill adults on intensive care: protocol for a randomised controlled feasibility trial
Source: Pilot Feasibility Stud. 2023 Jul 24;9:129. doi: 10.1186/s40814-023-01362-7 (PMC10364411; doi:10.1186/s40814-023-01362-7)
Supplement: Supplementary file 2 — Additional file 2: Appendix 1. Intervention arm protocol. [file 40814_2023_1362_MOESM2_ESM.docx]

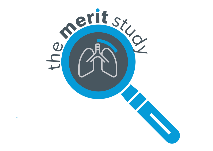


**Appendix 1: Intervention arm protocol**

Pre -procedure

- Check relevant equipment is ready. Note MI-E circuits may need to be double filtered for infection control purposes.
- Check session contraindications
  - PEEP>10
  - Haemodynamic instability: MAP <60 and >100, HR <50 and >130
  - Severe bronchospasm
- Complete routine airway clearance ensuring no secretions remain in the upper airways
- Note ETT insertion length at teeth and ensure the ETT cuff is adequately inflated
- Record pre-intervention observations into CRF

Procedure

| **Process step** | **Process detail** | **Considerations** |
| --- | --- | --- |
| 1 | Ensure closed suction in situ |  |
| 2 | Attach MI-E to the ETT with one hand holding the MI-E circuit and the other supporting the ETT (figure 1) |  |
| 3 | Deliver MI-E treatment cycles.  Update CRF re intervention detail (mode, pressures, timings, flow, repeats, other physiotherapy techniques used) | -patient positioning  -do you need to pre-oxygenate or entrain oxygen into the circuit (figure 2)  -starting pressures consider ≥PIP to optimise VT  -does the patient have recruitment needs?  -does the patient have a secretion load?  -are you likely to generate an expiratory flow bias?  -are other interventions required to facilitate/augment the expiratory flow bias? |
| 4 | Follow each cycle with suctioning of ETT. | It may be beneficial to insert the suction catheter into the ETT either before or during exsufflation to optimise secretion clearance |
| 5 | Rest on ventilator between cycles OR provide tidal volume breaths via MI-E | To prevent hyperventilation through blowing off CO_2_ and/or to minimise potential volutrauma |
| 6 | Repeat cycles until secretions are cleared | Regular re-Ax to determine treatment effectiveness |
| 7 | Monitor HR, BP and SpO2 throughout |  |

Post procedure

- Check ETT length and cuff pressure
- Re-auscultate
- Record post intervention observations as per protocol

Figure 1: set up of MI-E device to ETT

Figure 2: set up of MI-E to ETT to include entrained oxygen into the circuit
